# Bronchiectasis-COPD Overlap Syndrome: Role of Peripheral Eosinophil Count and Inhaled Corticosteroid Treatment

**DOI:** 10.3390/jcm12196417

**Published:** 2023-10-09

**Authors:** Grace Oscullo, Jose Daniel Gómez-Olivas, Marina Ingles, Sergio Mompean, Rosalia Martinez-Perez, Guillermo Suarez-Cuartin, David la Rosa-Carrillo, Miguel Angel Martinez-Garcia

**Affiliations:** 1 Respiratory Department, Hospital Universitario y Politécnico La Fe, 46026 Valencia, Spain; graceoscullo@gmail.com (G.O.); jdaniel365@gmail.com (J.D.G.-O.); azorinmarina@gmail.com (M.I.); sergiomompean@gmail.com (S.M.); rosaliamtezperez@gmail.com (R.M.-P.); 2 Instituto de Investigacion Sanitaria la Fe, 46026 Valencia, Spain; 3 Centro de Investigación Biomédica en Red (CIBERES), Instituto de Salud Carlos III, 28029 Madrid, Spain; 4 Pneumology Department, Hospital de Bellvitge, 08907 Barcelona, Spain; 5 Pneumology Department, Hospital de la Santa Creu i Sant Pau, 08041 Barcelona, Spain; david.rosa23@gmail.com

**Keywords:** overlap bronchiectasis-COPD, bronchiectasis, eosinophils, inhaled corticosteroids, mortality

## Abstract

Both chronic obstructive pulmonary disease and bronchiectasis are highly prevalent diseases. In both cases, inhaled corticosteroids (ICs) are associated with a decrease in exacerbations in patients with a high peripheral blood eosinophil count (BEC), but it is still not known what occurs in bronchiectasis-COPD overlap syndrome (BCOS). The present study aimed to assess the effect of ICs on various outcomes in patients with BCOS, according to BEC values. We undertook a post-hoc analysis of a cohort of 201 GOLD II-IV COPD patients with a long-term follow-up (median 74 [IQR: 40–106] months). All participants underwent computerized tomography and 115 (57.2%) had confirmed BCOS. A standardized clinical protocol was followed and two sputum samples were collected at each medical visit (every 3–6 months), whenever possible. During follow-up, there were 68 deaths (59.1%), and the mean rate of exacerbations and hospitalizations per year was 1.42 (1.2) and 0.57 (0.83), respectively. A total of 44.3% of the patients presented at least one pneumonic episode per year. The mean value of eosinophils was 402 (112) eosinophils/µL, with 27 (23.5%), 63 (54.8%), and 25 patients (21.7%) presenting, respectively, less than 100, 101–300, and more than 300 eosinophils/µL. A total of 84 patients (73.1%) took ICs. The higher the BEC, the higher the annual rate of exacerbations and hospitalizations. Patients with less than 100 eosinophils/µL presented more infectious events (incident exacerbations, pneumonic episodes, and chronic bronchial infection via pathogenic bacteria). Only those patients with eosinophilia (>300 eosinophils/µL) treated with ICs decreased the number (1.77 (1.2) vs. 1.08 (0.6), *p* < 0.001) and the severity (0.67 (0.8) vs. 0.35 (0.5), *p* = 0.011) of exacerbations, without any changes in the other infectious outcomes or mortality. In conclusion, ICs treatment in patients with BCOS with increased BEC decreased the number and severity of incident exacerbations without any negative influence on other infectious outcomes (incidence of pneumonia or chronic bronchial infection).

## 1. Introduction

Both chronic obstructive pulmonary disease (COPD) and bronchiectasis are highly prevalent inflammatory diseases of the airways [1,2,3,4]. The fundamental endotypic substrate of both is the presence of mixed inflammation with a predominance of neutrophils and a variable infiltration by eosinophilic and mononuclear cells [5,6,7,8,9], which have been associated in both diseases with chronic infection by potentially pathogenic microorganisms [10,11,12,13,14,15] and the number and severity of exacerbations [16,17,18,19,20,21,22].

In recent years, it has been observed in both diseases that the peripheral blood eosinophil count (BEC) correlates to a moderate extent with eosinophilic infiltration in the bronchial mucosa, even in individuals without asthma [2,3,21,22]. The BEC seems to correlate well with some clinical aspects and with the response to some treatments in both diseases. Thus, a BEC greater than 300 eosinophils/µL implies a positive response to inhaled corticosteroids (ICs) through a significant decrease in the number and severity of exacerbations, even though it is associated with more clinically severe forms of both diseases [2,3,21,22]. However, a BEC below 50–100/µL is associated with a lack of response to ICs, as well as a higher percentage of adverse effects, including pneumonic processes [2,3,21,22]. Furthermore, a BEC value of less than 50–100 eosinophils/µL in bronchiectasis is associated with a greater severity of the disease [21,22].

ICs are drugs with a significant anti-inflammatory and also immunosuppressive capacity, so their use can cause an excess of respiratory infections by potentially pathogenic microorganisms, especially bacteria [23,24]. This situation may be even more frequent in individuals who already suffer from a chronic bronchial infection (CBI)—a situation that may occur in up to 30% of patients with severe COPD and in more than 70% of those with bronchiectasis throughout their natural history—caused in many cases by very virulent pathogenic microorganisms such as *Pseudomonas aeruginosa* [25,26].

COPD and bronchiectasis share similar pathophysiological mechanisms. It is currently thought that up to 50% of individuals with severe COPD may present with bronchiectasis [27], and that up to 10% of patients with bronchiectasis may present with COPD [28]. This phenomenon has come to be called BCOS (bronchiectasis-COPD overlap syndrome) [29]. In this group of patients, the role of BEC in several important outcomes is unknown, as is the effect of treatment with ICs on the number and severity of exacerbations, according to the BEC value. Taking into account the positive effect of ICs in preventing exacerbations and other important outcomes in both COPD [2,3] and bronchiectasis [21,22] patients with peripheral eosinophilia, we hypothesize that similar effects will be seen in BCOS patients. Therefore, the main objective of this study was to analyze, in a group of patients with BCOS and a long-term follow-up, the relationship between the initial BEC value and some outcomes of interest, including mortality, changes in the microbiological profile, and the number and severity of exacerbations, as well as the response to treatment with ICs.

## 2. Methods

### 2.1. Study Design, Participants, and Ethics

This was a post-hoc analysis of a cohort of 201 COPD patients recruited between January 2004 and February 2007 in two specialized out-patient clinics in Spain [30]. The included patients had moderate-to-very-severe COPD (GOLD II-IV), a cumulative smoking exposure > 10 packs/year, and the capacity to provide spontaneous valid sputum samples during follow-up. For the purpose of this study, only those with clinically active bronchiectasis (BCOS) were included [30]. The exclusion criteria included allergic bronchopulmonary aspergillosis (ABPA), asthma, and treatment with anti-eosinophil biological treatments or systemic corticosteroids. Asthma was excluded following international guideline recommendations based on the lack of typical symptoms and negative complementary tests in case of doubt.

Patients were extensively characterized at recruitment and visited every 3–6 months, depending on their individual clinical condition. Measurements were always obtained during clinical stability, at least 6 weeks after any exacerbation. The study was approved by the Ethics Committee of the Hospital General Universitario de Valencia (ID 2003-0089) and all participants signed their informed consent.

### 2.2. Clinical Characterization

As detailed elsewhere [31], we used structured questionnaires to record the symptoms (dyspnoea [mMRC] and sputum production), baseline and follow-up smoking status (current vs. former), cumulative exposure (pack/year), exacerbations and/or comorbidities in the past or the follow-up (Charlson comorbidities index or other relevant past medical history), baseline blood cell counts (including baseline BEC), general biochemistry, and current medical treatment (including IC treatment). COPD exacerbations were defined as an increase in two or more cardinal symptoms (dyspnoea, sputum quantity, and/or purulence) treated with corticosteroids and/or antibiotics (moderate exacerbation) or hospitalization (severe exacerbation) [32]. Forced spirometry was determined following international standards and the reference values were those of Roca et al. [33].

We recorded the vital status at each clinical visit. Cause of death was obtained from official death certificates or from hospital medical records. This information was complemented by a review of outpatient medical records, computerized databases, and contact with the patient’s relatives or primary care physician.

### 2.3. High-Resolution CT Scan (HRCT Scan)

HRCT scans were obtained in both centers in all patients included in the study, using a 16-slice multidetector CT scanner (Bright Speed 16, General Electric, Fairfield, CT, USA) during a fully suspended inspiration in the supine position and using a thin-section technique (1 mm collimation at 10 mm intervals). A high spatial frequency algorithm was used for image reconstruction. The images were obtained without any injection of contrast material. The small cylindrical bronchiectasis visible in only a single pulmonary segment was not considered. The HRCT scans were independently interpreted in each participating center by a radiologist with at least 10 years of experience in diagnosing bronchiectasis, masked to the patients’ basal characteristics. Any differences in the readings were resolved via consensus. The diagnosis of bronchiectasis was based on the criteria recently published by Aliberti et al. as a typical radiological image and associated clinical picture, especially cough with sputum production and/or exacerbations of the infectious profile. The radiological extent and type of bronchiectasis were evaluated according to the number and location of the pulmonary lobes and segments affected (with the lingula considered an independent lobe), and the presence of cystic bronchiectasis or central bronchiectasis [30].

### 2.4. Microbiology

Patients were asked to collect two sputum samples at home using a sterile technique for every medical visit and to return them to the hospital within less than 3 h of collection for microbiological analysis. Sputum samples were considered valid if they contained <25 squamous epithelial cells/low-powered field and >than 25 leukocytes/high-powered field. Gram stained and homogenized were performed, as well as serial diluted secretions were plated on chocolate, blood, Saboureaud, and McConkey agar. Sputum cultures were expressed as CFU per milliliter. A threshold of ≥10^3^ CFU was considered positive for pathogenic microorganisms, including *Streptococcus pneumoniae*, *Haemophilus influenza*, *Staphylococcus aureus*, *Moraxella catarrhalis*, *Pseudomonas aeruginosa*, *Klebsiella pneumoniae*, and other Gram-negative and -positive rods.

### 2.5. Data Analysis

**Figure 1:**
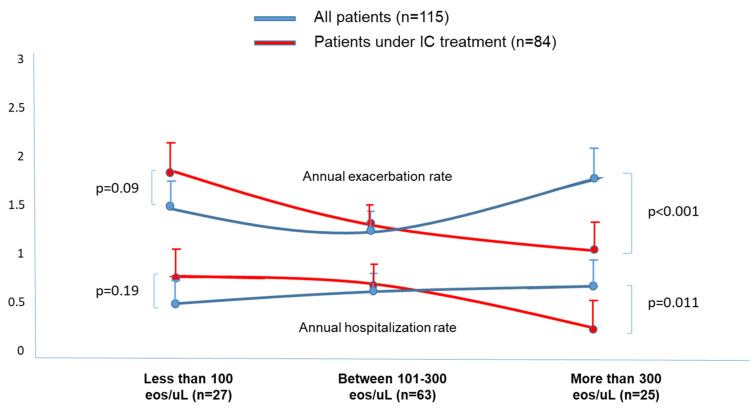
Annual exacerbation and hospitalization rates in the groups of patients, according to the blood eosinophil count in both groups (with and without inhaled corticosteroid treatment). Those patients under inhaled corticosteroid treatment and more than 300 eosinophils/µL presented a significant decrease in the annual exacerbation rate compared with patients without eosinophilia. Eos: eosinophils.

**Figure 2:**
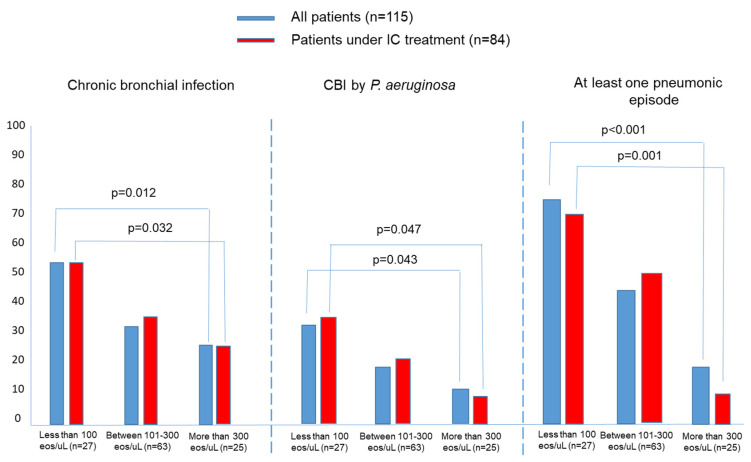
Prevalence of patients with chronic bronchial infection, chronic bronchial infection by *Pseudomonas aeruginosa* and at least one pneumonia episode during follow-up in the groups of patients, according to the blood eosinophil count in both groups (with and without inhaled corticosteroid treatment). Patients with less than 100 eosinophils/µL presented more chronic bronchial infection and pneumonic episodes than those with eosinophilia. CBI: Chronic bronchial infection; Eos: eosinophils.

The results were shown as a percentage in the case of dichotomous or qualitative variables, and the mean (standard deviation [SD]) or median (interquartile range [IQR]) in the case of quantitative variables depending on the distributions of the variables assessed via the Kolgomorov–Smirnov test. The participants were stratified into three groups, according to the baseline BEC value: less than 100 eosinophils/µL; between 101–300 eosinophils/µL; and more than 300 eosinophils/µL. Intergroup baseline variables (Table 1) as well as the relationship between the BEC value in the four groups and outcomes (Table 2) were compared by means of a one-way ANOVA test with Bonferroni correction or Chi-squared tests depending on the distribution of the variables. To compare the intragroup variables, a Student’s *t*-test was used (Figure 1). To compare the two proportions, a Chi-squared test is used in Figure 2. A two-sided *p* value < 0.05 was considered statistically significant. All analyses were performed using Stata v.11 or SPSS v. 21.

## 3. Results

Of the initial 201 COPD patients, 115 (57.2%) were diagnosed as having bronchiectasis (BCOS). The mean age was 71.4 (8.5) years (92.5% male) with a post-bronchodilator FEV1 pred% of 45.4% (12.8) and a smoking habit of 62.1 (31.9) pack.years; 60.9% were being treated with triple therapy and 73% with double bronchodilator therapy (LABA + LAMA). Thirty-one patients (26.9%) were not taking ICs.

The median follow-up was 74 (IQR: 40–106) months. During follow-up, there were 68 deaths (59.1%), and the mean rate of exacerbations and hospitalizations per year was 1.42 (1.2) and 0.57 (0.83), respectively. A total of 44.3% of the patients presented at least one pneumonic episode per year. The median number of medical visits during follow-up was 21.6 (IQR: 10.3–25.1) and the median number of valid sputum samples per patient during follow-up was 18.9 (IQR: 10.1–23.4).

The mean value of eosinophils was 402 (112) eosinophils/µL, of which 27 (23.5%), 63 (54.8%), and 25 (21.7%) had less than 100, between 101–300, and more than 300 eosinophils/µL, respectively. The characteristics of the three groups of patients, according to the BEC, are shown in Table 1. No significant differences were observed in the general, clinical, functional, and therapeutic variables between groups.

### 3.1. Blood Eosinophil Count and Follow-Up Variables

Table 2 shows how, in the group of patients (*n* = 115), those who presented a higher number of eosinophils (more than 300/µL) had a higher annual rate of exacerbations compared to patients with less than 300 eosinophils/µL; 1.77 (1.2) vs. 1.27 (1.1) and 1.48 (1.1); *p* = 0.031); there was also a higher number of hospitalizations for patients with less than 100 eosinophils/µL, compared to those with less than 50/µL: 0.67 (0.8) vs. 0.48 (0.7); *p* = 0.041). The results referring to the exacerbations/hospitalizations are also represented in Figure 1. However, it was the patients with a decreased number of eosinophils (<100 eosinophils/µL) who presented more infectious events, either in the form of CBI compared to those with more than 300 eosinophils/µL (51.8% vs. 24%; 0.012), or in the form of CBI via PA compared to those with more than 100 eosinophils/µL (33.3% vs. 17.4% and 12%); *p* = 0.043, and a higher percentage of patients with at least one pneumonic process during follow-up compared to those with more than 100 eosinophils/µL (74.1 vs. 44.4% and 12%; *p* < 0.001). The results related to infectious outcomes are also represented in Table 2. There were, however, no significant differences regarding the mortality between the groups analyzed.

### 3.2. Effects of Treatment with ICs According to the BEC

Figure 1 shows how, in those patients treated with ICs (*n* = 84), there was a significant decrease in the annual rate of exacerbations and hospitalizations in the group with more than 300 eosinophils/µL (1.77 (1.2) vs. 1.08 (0.6), *p* < 0.001), respectively. However, there were no significant differences in the rest of the groups, according to the BEC value, although it is worth noting that in those patients with less than 100 eosinophils/µL, there was a non-significant tendency towards an increase in the annual rate of exacerbations and hospitalizations. (1.48 (1.1) vs. 1.71 (1.1); *p* = 0.09) and (0.48 (0.7) vs. 0.69 (0.8), *p* = 0.19), respectively. No significant changes were observed (Figure 2) during the follow-up between patients who took and did not take ICs in terms of the proportion of CBI due to any pathogenic microorganism, due to *Pseudomonas aeruginosa*, or due to the presence of at least one pneumonic process; similarly, no changes were observed in the mortality rate (Table 2).

## 4. Discussion

According to our results, those patients with BCOS and eosinophilia (>300 eosinophils/µL) presented a significantly higher annual rate of exacerbations and non-pneumonic hospitalizations during their follow-up, while those with a decreased number of BEC (<100 eosinophils/µL) presented an increased probability of CBI (including PA) and pneumonic processes. Treatment with ICs significantly reduced the annual incidence of exacerbations and non-pneumonic hospitalizations only in the group with peripheral eosinophilia, although without modifying the microbiological profile, the number of pneumonic processes, or the mortality rate.

Current COPD guidelines do not usually consider the presence of bronchiectasis and/or CBI due to pathogenic microorganisms when assessing the effect of ICs, their adverse effects, or the risk of exacerbations or pneumonia, but are instead usually based on the value of BEC [2,3,34]. However, it is known that up to 50% of patients have severe COPD present bronchiectasis [25] and that between 30–50% of patients with COPD presented isolates of pathogenic microorganisms throughout their natural history [27]. Considering the potent anti-inflammatory and immunosuppressive actions of ICs, the presence of pathogenic microorganisms in the airways of COPD patients seems potentially important [35].

In both patients with COPD and those with bronchiectasis, the interaction between the level of peripheral eosinophils, the existence of bronchial infection by pathogenic microorganisms, treatment with ICs, and the risk of exacerbations or pneumonic processes as a consequence of this treatment are all complex [35]. A recent study showed that, in patients with moderate-severe COPD, the risk of future pneumonia in those patients treated for ICs depended both on the level of peripheral eosinophils and on the additional existence of CBI due to pathogenic microorganisms. Thus, although the risk of pneumonia was increased in patients with less than 100 eosinophils/µL, this risk increased more in the presence of CBI, especially in those patients treated with ICs. However, in patients with >100 eosinophils/µL, the risk of pneumonia was only increased in the context of CBI and not in that of treatment with ICs [36].

In the case of patients with bronchiectasis, the use of ICs is generally not indicated, except in patients with asthma and eosinophilic COPD, but, nevertheless, these drugs are clearly being overused [37,38,39]. There are no studies on the role of ICs in bronchiectasis with CBI, but one recent study showed that, in patients with bronchiectasis and peripheral eosinophilia (at least 300 eosinophils/µL), treatment with ICs significantly reduced the number and severity of exacerbations, even in the absence of asthma (although this study did not examine the interaction of a CBI in this association) [22].

The third scenario would be that of the patient with BCOS, the subject of the present study. Our results bring up some interesting considerations: (1) Those patients with BCOS and peripheral eosinophilia showed a higher annual rate of exacerbations and non-pneumonic hospitalizations during their follow-up. This situation had already been observed in both patients with COPD [40] and those with bronchiectasis [23]. However, in the present study, the presence of less than 100 eosinophils/µL was not associated with a greater number of exacerbations, even though this had been the case in previous studies in patients with bronchiectasis. (2) Those patients with <100 eosinophils/µL presented a greater number of CBI due to pathogenic microorganisms, as well as pneumonic processes, during their follow-up. This situation had already been observed in patients with both COPD and bronchiectasis [2,3,21,22]. It is possible that, due to the known antiviral and bactericidal effect of eosinophils, a drop in their numbers could induce an increase in the number of both viral and bacterial respiratory infections [41]. (3) Treatment with ICs, as occurs with patients with COPD and with bronchiectasis [2,3,22], significantly reduced the number and severity of exacerbations only in BCOS patients with eosinophilia, without any changes in the other groups, according to the BEC. It may be noteworthy that treatment with ICs induced a non-significant tendency towards an increase in the annual rate of exacerbations and hospitalizations in patients with less than 100 eosinophils/µL (which was not observed in patients with a number of eosinophils/µL between 101–300). In any case, the latter results must be taken with caution, since a type 2 statistical error may have occurred due to the small number of patients included.

However, paradoxically, there were no changes in the microbiological profile or the risk of pneumonic processes during follow-up, regardless of the value of peripheral eosinophils. This finding is not easy to explain and possibly reflects the complex relationship between COPD, bronchiectasis, peripheral eosinophils, pneumonia, and exacerbations. It is possible that the existing discrepancy between the reduction in the rate of exacerbations produced by ICs in the presence of eosinophilia, but not in pneumonia or CBI, may be due to a predominance of the anti-inflammatory action over the immunosuppressive action in the presence of eosinophilia [41]. Another hypothesis that needs to be assessed would be the different origin (viral or bacterial) of these infectious processes. CBI is, by definition, a bacterial process, while exacerbations and pneumonia in patients with COPD and bronchiectasis may also have a viral origin (especially in patients with COPD). Thus, it is possible that the influence of the effect of ICs, and its modulation by the presence of peripheral eosinophilia, is determined via the microbiological profile (either viral or bacterial) that caused the infectious process. Unfortunately, we do not have sufficient data to analyze this hypothesis, but it opens up an interesting field of research for future studies.

A very recent retrospective study carried out using electronic health care records in the USA has been the only one to analyze the risk of hospitalization for pneumonia in patients with BCOS and treatment with ICs. This study concluded that the use of ICs did not further increase the already increased risk of bronchiectasis in patients with BCOS, but that in the subgroup study, this risk only appeared in those patients who were taking ICs and had less than 300 eosinophils µL and not in those with eosinophilia [42]. In this respect, the results of this study concur with ours in not observing an increase in the risk of pneumonia after taking ICs, although it was observed in patients with a reduction in the number of eosinophils, regardless of whether or not they took ICs.

One of the strengths of this study is that it was carried out on patients with BCOS who were very well characterized from the clinical and microbiological point of view, followed up over a long period of time from a database designed for the collection of patients with COPD and bronchiectasis. Among the limitations, it is important to highlight the limited number of included patients; that we do not have data on the accumulated doses of ICs, which may be an important factor; and that these results cannot be extrapolated to the general group of patients with BCOS, but only to those with moderate-to-severe COPD with productive cough, although in the vast majority of cases in which bronchiectasis is observed in patients with moderate or severe COPD, there is an increase in sputum production. Finally, we performed semi-quantitative techniques to analyze the microbiological profile of the sputum samples. Moreover, techniques to analyze the lung microbiome was not included.

In short, the relationship between COPD, bronchiectasis, treatment with ICs, risk of pneumonia, rate of exacerbations, and BEC is complex in patients with BCOS. As occurs in patients with COPD or bronchiectasis separately, peripheral eosinophilia is a marker of a good response to treatment with ICs in those with BCOS, without any negative influence of this treatment on the microbiological profile and its consequences (exacerbations or pneumonia). This negative impact was seen, however, in those patients with a reduced number of peripheral eosinophils. Since eosinophilic inflammation and bronchial infection are potential treatable traits [43,44] related to a higher number of exacerbations which impact the prognosis in both bronchiectasis and COPD [45,46], it seems important to monitor the number of peripheral eosinophils in patients with BCOS. New studies on the effect of ICs [23,47] and antieosinophils biologics are needed [48,49,50], as well as the relationship between BEC and the microbiological profile using techniques to analyze the lung microbiome.

## Figures and Tables

**Table 1:** Baseline characteristics based on blood eosinophil counts.

Variables	Less than 100 Eosinophils/µL	Between 101–300 Eosinophils/µL	More than 300 Eosinophils/µL	*p* Value (ANOVA)
	*n* = 27 (23.5%)	*n* = 63 (54.8%)	*n* = 25 (21.7%)	
Age, yrs.	72.2 (9.2)	71.6 (8.5)	70.6 (8.3)	0.801
Males, %	89%	94%	96%	0.586
Body mass index, kg/m^2^	27.2 (3.9)	25.8 (4.8)	27.7 (5.6)	0.182
Smoking habit, pack.years	55.5 (29.2)	66.3 (30.4)	58.6 (37.7)	0.285
Charlson index	2.6 (1.4)	2.6 (1.5)	2.2 (1.4)	0.481
Dyspnoea (MRC)	1.9 (0.9)	1.6 (0.9)	1.6 (0.9)	0.356
Daily sputum production, %	77.8%	74.6%	64%	0.567
Purulent sputum, %	22.2%	25%	33.3%	0.858
Time from symptoms	14 (16.6)	13.4 (10.7)	10.7 (10.6)	0.578
Post-bd FEV1, % ref.	46.6 (13.7)	45.1 (13.1)	45.9 (11.7)	0.879
FEV1/FVC, %	52.7 (13.8)	48.8 (13.2)	52.7 (11.9)	0.291
Fibrinogen, mg/dL	327 (145)	430 (172)	338 (221)	0.132
CRP, IU/mL	8.2 (6.9)	8.1 (8.5)	9.1 (10)	0.947
Long-acting bronchodilators, %	97%	98%	96%	0.956
Inhaled corticosteroids, %	64%	69%	66%	0.879
Triple therapy, %	51.8%	58.7%	64%	0.676
Long-term antibiotics	26%	23%	15%	0.064
Macrolides	28%	25.4%	16%	0.086

**Table 2:** Effect of inhaled corticosteroid treatment on the annual number of exacerbations, hospitalizations, incidence of chronic bronchial infection, pneumonia, and all-cause death.

Variables	Less than 100 Eosinophils/µL	Between 101–300 Eosinophils/µL	More than 300 Eosinophils/µL	*p* Value (ANOVA)
	*n* = 27 (23.5%)	*n* = 63 (54.8%)	*n* = 25 (21.7%)	
Annual exacerbation rate − All patients − Under ICs treatment	1.48 (1.1)	1.27 (1.1)	1.77 (1.2)	0.031 *
	1.71 (1.1)	1.31 (1.1)	1.08 (0.6)	0.011 **
Annual hospitalization rate − All patients − Under ICs treatment	0.48 (0.7)	0.58 (0.9)	0.67 (0.8)	0.041 **
	0.69 (0.8)	0.61 (0.9)	0.35 (0.5)	0.033 **
Chronic bronchial infection − All patients − Under ICs treatment	51.8%	31.7%	24%	0.012 **
	50%	34%	24.3%	0.032 **
CBI by *P. aeruginosa* − All patients − Under ICs treatment	33.3%	17.4%	12%	0.043 ***
	35%	20%	7.1%	0.047 ***
At least one pneumonia − All patients − Under ICs treatment	74.1%	44.4%	12%	<0.001 ***
	70%	50%	7.1%	0.001 ***
Death − All patients − Under ICs treatment	59.2%	57.1%	64%	0.844
	55%	64%	57.1%	0.757

* More than 300 eosinophils/µL vs. 101–300 eosinophils/µL. ** Less than 100 eosinophils/µL vs. more than 300 eosinophils/µL. *** Less than 100 eosinophils/µL vs. 101–300 and more than 300 eosinophils/µL. ICs: Inhaled corticosteroids; CBI: Chronic bronchial infection; *P. aeruginosa. Pseudomonas aeruginosa.*

## Data Availability

Data is available under reasonable request and after evaluation by the scientific committee of the study.
